# Internet of Things to Explore Moment 2 of “WHO My Five Moments” for Hand Hygiene

**DOI:** 10.3389/fdgth.2021.684746

**Published:** 2021-10-21

**Authors:** Olga Florea, Jeremy Gonin, Hervé Tissot Dupont, Jean Charles Dufour, Philippe Brouqui, Sophia Boudjema

**Affiliations:** ^1^Aix Marseille Université, IRD, MEPHI, IHU-Méditerranée Infection, Marseille, France; ^2^AP-HM, IHU-Méditerranée Infection, Marseille, France; ^3^Aix Marseille Université, AP-HM, INSERM, IRD, SESSTIM, Hop Timone, BioSTIC, Marseille, France

**Keywords:** hand hygiene (disinfection), IoT - internet of things, five moments for hand hygiene, nosocomial infections, catheter - complications

## Abstract

**Background:** Electronic hand hygiene surveillance systems are developing and considered to be more reliable than direct observation for hand hygiene monitoring. However, none have the capability to assess compliance in complex nursing care.

**Materials and Methods:** We combined two different technologies, a hand hygiene monitoring system (radiofrequency identification, RFID) and a nursing care recorder at the bedside, and we merge their data to assess hand hygiene performance during nursing. Nursing tasks were classified as standard task procedures or aseptic task procedures corresponding to moment 2 among the five moments for hand hygiene recommended by the WHO. All statistical analyses were performed using R, version 3.6.2. For mixed models, the package “lme4” was used.

**Results:** From the merged database over the 2-year study period, 30,164 nursing tasks were identified for analysis, 25,633 were classified as standard task procedures, and 4,531 were classified as aseptic task procedures for nursing care. Hand disinfection with an alcohol-based solution was not detected with our system in 42.5% of all the recorded tasks, 37% of all the aseptic task procedures, and 47.1% of all the standard task procedures for nursing (*p* = 0.0362), indicating that WHO moment 2 was not respected in 37% of mandatory situations.

**Conclusion:** Using a combination of different technologies, we were able to assess hand hygiene performance in the riskiest circumstances.

## Introduction

It is generally believed that hand hygiene reduces the prevalence of hospital-acquired infections (HAIs), and that inadequate hand hygiene is one of the main risk factors for infection (1–4).

The risk of transmission of microbes from the hands of healthcare workers (HCWs) to patients has been studied extensively in peripheral venous catheters (PVCs), which have long been associated with infection (4–6).

Appropriate hand disinfection prior to PVC insertion significantly reduces the incidence of infection (5–8). Direct observation is the current gold standard to appreciate compliance to hand hygiene. A model of transmission identifying moments at which healthcare workers (HCWs) are at risk for transmission has been suggested (9). This model was used to develop “My Five Moments for Hand Hygiene” (10, 11). Alcohol-hand-rub (AHR) with an alcohol-based solution before moments 1 and 2 is suggested to prevent the transmission of infection to patients, whereas AHR after moments 3, 4, and 5 is suggested to protect the environment of patients and the transmission of infection to other patients. Moment 2 of the My Five Moments by the WHO is defined as hand disinfection before an aseptic task procedures (12). While Moment 2 appears as a very important clue to cross transmission, its monitoring by direct observation is difficult as events are relatively infrequent compared with moments 1 and 5.

To bypass this difficulty, we have developed a tool to record the task of a nurse at the bedside of the patient that we called patient smart reader (PSR) (13). This personal digital assistant allows to record nurses care at the bedside and send data in a centralized database connected to hand hygiene monitoring system and available for nurse's feedback in real time.

An automated hand hygiene monitoring system has recently been developed with the aim to replace direct observational monitoring, because it avoids the influence of the Hawthorne effect and appears to be more reliable (14–16). Since 2012, we have developed an automated hand hygiene monitoring system and used it as a part of the HAI Management at the University Hospital Institute Méditerranée Infection (IHU-MI) (17–19). However, to our knowledge, there are no automated hand hygiene monitoring systems that allow for the evaluation of hand hygiene before an aseptic task procedure (moment 2), except for remote video-based surveillance systems, which are time-consuming and not cost-effective (20, 21).

This study aimed to evaluate the hand hygiene of nurses before an aseptic task procedure (moment 2) using merge data provided by our automatic hand hygiene monitoring system coupled with the PSR. The hand hygiene monitoring system provided alcoholic hand rub use and entry/exit of the bedroom, and the PSR provided information on the nature of the nursing task recorded during care. This way, we investigated if nurses performed ARH during care and for which kind of care.

## Materials and Methods

### Electronic Survey

For this study, we used a combination of data from two different electronic data capture (EDC) systems (see functional schema in Supplementary Material). An EDC system is a computerized system designed for the collection of clinical data in electronic format for use mainly in human clinical trials. The first EDC system was a hand hygiene automated electronic system named MediHandTrace^®^ (MHT), which is an RFID (13.56 MHz)-based personal identifying tracking system that records compliance to AHR for each identified HCW. This system was deployed in a 25-bed infectious disease ward in Marseille, France (18). MHT detects the movements of HCWs by tracking chips that are placed in the shoes of HCWs; RFID signals are emitted by an antenna placed on the floor at the entrance of the bedroom. When an HCW enters the room by opening the door, the device is triggered, and a set of signals are captured and stored in a server at the following time points: [0] when the door opens, [1] when the HCW walks near the antenna, [2] when the HCW walks out of the range of the antenna, and [8] when AHR is performed within or [10] more than 8 s after the HCW enters the room. The second EDC system was a handy personal digital assistant with a barcode reader named the Patient Smart Reader (PSR), a device that allows to record, at patient bedside, nursing tasks. This tool allows for integration of recorded data within the SQL database stored in the PSR, and are then synchronized with a desktop- or server-based database. It allows nursing care tasks and vital signs of patients to be recorded by HCWs in real time during the provision of care (note that an HCW can record the action before or after he/she performs it) (13). This is an important limitation, which explains that only the lack of compliance to AHR can be explored. If AHR is performed, we cannot identify if this is before (good) or after (bad) the task. To record a task, an HCW must identify himself or herself, identify the patient, and identify the task by scanning the barcode affixed to the wall next to the bed. Both systems (MHT and the PSR) are time-synchronized and send the collected data on the same server using a unique ID for each HCW. Together, these two systems enable hand hygiene assessment performed at the time of a specific nursing task.

### Selection of Variables

The nursing tasks that were explored are listed in Table 1. They were classified as standard task procedures (STPs) or aseptic task procedures (ATPs) corresponding to moment 2 for hand hygiene. Each act recorded in a designated aseptic task should be associated with one AHR (hand hygiene opportunity). The lack of compliance to AHR is defined by the ratio of no AHR/number of acts in the designated task.

**Table 1:** Ratio of lack of compliance to hand hygiene among 30,164 categorized nursing tasks as surveyed by electronic monitoring system.

**Nursing task**	NO AHR^*^	**Total acts**	**(%)**	**Task category**
Final household	139	200	69.5	STP
Patient departure	1,076	1,729	62.2	STP
Meal stimulation	92	177	52.0	STP
Vital signs recording	5,461	10,722	50.9	STP
Drinking stimulation	152	304	50.0	STP
Care of eschars	59	119	49.6	ATP
Meal aid	115	235	48.9	STP
Penile case pose	13	27	48.1	STP
Diet collation	12	25	48.0	STP
Prevention of eschars	468	1,003	46.7	STP
Refurbishment of the bed	525	1,145	45.9	STP
Layer Pose	646	1,416	45.6	STP
Eyes care	163	358	45.5	STP
Drink aid	129	285	45.3	STP
Mouth care	183	405	45.2	STP
Dressing maintenance	150	334	44.9	ATP
Meal installation	164	367	44.7	STP
Changing diaper	565	1,272	44.4	STP
Chair installation	72	163	44.2	STP
Exchange urine pouch	32	75	42.7	STP
Removal dressing	54	127	42.5	ATP
Removal penile case	14	33	42.4	STP
Patient toilet	234	569	41.1	STP
Emptying urine pouch	93	227	41.0	ATP
Urinary catheter monitoring	168	419	40.1	ATP
Blood Catheter monitoring	653	1,753	37.3	STP
Blood Catheter Removing	144	391	36.8	ATP
Sub cutaneous medication	239	659	36.3	ATP
Per os medication	867	2,391	36.3	STP
Urinary catheter insertion	17	47	36.2	ATP
Dressing Pose	58	163	35.6	ATP
Blood infusion transfusion	8	23	34.8	ATP
Blood culture	41	118	34.7	ATP
Intravenous medication	360	1,042	34.5	ATP
Blood test collection	180	524	34.4	ATP
Blood Catheter insertion	93	292	31.8	ATP
Urinary catheter removing	14	46	30.4	ATP
Daily housekeeping	288	953	30.2	STP
Penile case watch	7	26	26.9	STP
Total		30,164		

*Lack of compliance is defined as no alcoholic hand rub (NO AHR)/ number of dedicated acts recorder with PSR*.

* *No alcoholic hand rub for task recording*.

*STP, Standard task procedure; ATP, Aseptic task procedure*.

### Data Analyses

The data used for the study were extracted from the raw data (MHT database and PSR database) collected from January 11, 2017 to January, 11, 2019. The lack of compliance during ATPs was compared with the lack of compliance during STPs. Both systems have been used in the ward for years, and HCWs were invited to participate in the study as part of their natural nursing routine. No specific training was given before the study.

### Statistical Analysis

All the statistical analyses were performed using R, version 3.6.2. For the mixed models, the package “lme4” was used. For all the statistical tests, the alpha risk level was set to 5%, and a bilateral alternative hypothesis (two-sided test) was used, except when the Kolmogorov–Smirnov test was performed with a unilateral alternative hypothesis (one-sided test). The lack of compliance with hand hygiene was compared across durations by the chi-square test.

In a previous study, we reported that the use of an automated electronic surveillance system generated a large amount of data for which the bias in the relationship between HCW activity and HCW performance with AHR was needed to be corrected using a multilevel multivariate logistics model (18). To better control the link between hand hygiene compliance and the type of nursing care, the following generalized linear mixed models were formulated:


$$\begin{array}{l} M_{0}\;:\;logit\left(P\left(Y_{ij}=1\right)\right)=\beta_{0} \end{array}$$

$$\begin{array}{l} M_{0r}\;:\;logit\left(P\left(Y_{ij}=1\right)\right)=\beta_{0}+\;b_{0i}b_{0i}\sim N\left(0,\sigma_{0}^{2}\right) \end{array}$$

$$\begin{array}{l} M_{1}\;:\;logit\left(P\left(Y_{ij}=1\right)\right)=\beta_{0}+\beta_{1}X_{ij} \end{array}$$

$$\begin{array}{l} M_{1r}\;:\;logit\left(P\left(Y_{ij}=1\backslash b_{0i}\right)\right)=\beta_{0}+\beta_{1}X_{ij}+b_{0i}b_{0i}\sim N\left(0,\sigma_{0}^{2}\right) \end{array}$$

where Y is the hand hygiene compliance variable (Y = 0 if AHR is not used and Y = 1 if AHR is used) and X is the nursing task risk variable (X = 0 if the nursing task is an STP and X = 1 if the nursing task is an ATP). The index i represents the HCW level, j represents the nursing care level, and *Y*_*ij*_ represents hand hygiene compliance. The different models were compared using several methods. The Akaike inference criterion (AIC) was used when the models were not nested. For the nested models, comparisons were made with the likelihood ratio test corrected for the comparison between two mixed models. For the models with random effects, the intraclass correlation coefficient (ICC) was used to measure the percentage of variance in AHR use attributable to HCW level. The ICC was estimated based on assumptions for binary variables, and the variance attributable to the HCW level was divided by the total estimate variance. For the models without random effects, the estimations were maximum-likelihood estimations, and for the mixed models (with random effects), the maximum-likelihood estimation method with Laplace approximation was used. Finally, to investigate when AHR was performed within the care sequence, the distribution of hand hygiene within the care sequences was compared between the STPs and ATPs for nursing. The duration of each care sequence was split into 100 intervals of the same length, and the significance was tested by the Kolmogorov-Smirnov test with a unilateral alternative hypothesis (Supplementary Material). The data set is available upon request to the corresponding author.

### Ethics

To ensure the anonymity of the data analyzed, a random number was assigned to the data from each participant included in the database. All the procedures for this study were approved by the Ethics Committee of our institution (N° 2016-018). Before the study, the HCWs were informed of the details of the study and they gave their consent to be monitored by the automated systems.

## Results

Among 39 nursing procedures, 24 were classified as standard task procedures (STPs) and 15 were classified as aseptic task procedures (ATPs) for which ARH before the procedure is mandatory (Moment 2). The most frequent procedures, such as recording of vital signs, per os medication, and layer pose were STPs. Among the ATPs, blood catheter monitoring, intravenous medication, and blood test collection were the most frequent. Our system identified the HCW in the patient room for 30,164 nursing tasks procedures during the 2-year study period. Of the 30,164 tasks, 25,633 were classified as requiring STPs for nursing, and 4,531 were classified as requiring ATPs for nursing (Table 1).

**Figure 1:**
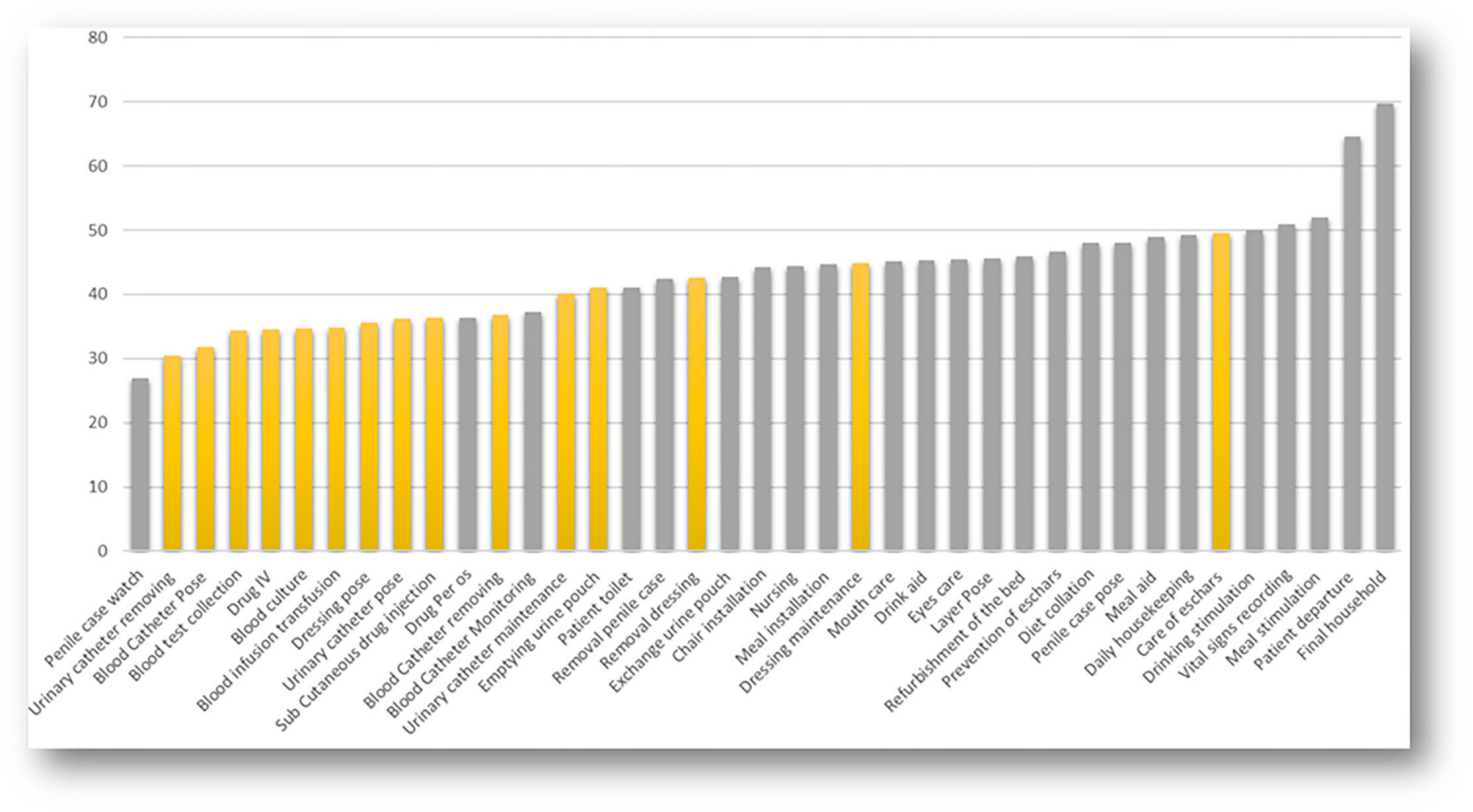
Distribution of lack of compliance (%) to hand hygiene in aseptic task procedure (WHO Moment 2) vs. standard task procedure nursing. Hand hygiene is better performed when the nursing is in the category aseptic task (yellow) compare to standard precaution (gray) that were 1,678 (37%)/12,070 (47.1%) *p* < 2.2 E-16.

**Figure 2:**
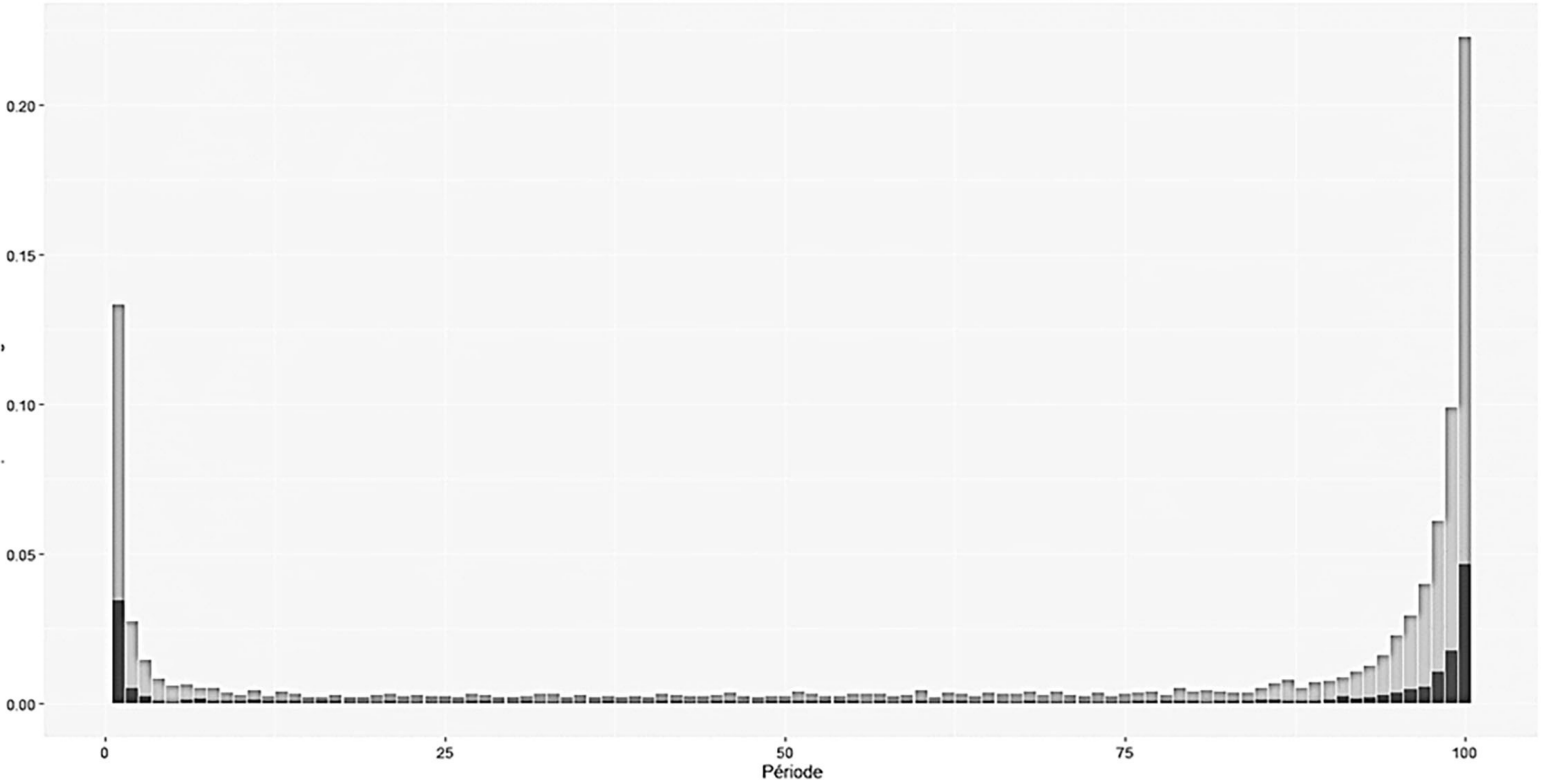
Distribution during the nursing task (from room entry to room exit) of alcoholic hand rub among 16,416 nursing tasks, 12,759 classified standard task procedures STP (in gray) and 2,853 aseptic tasks procedures ATP (in black). Hand hygiene is more frequently performed at the end of the task when standard task procedure nursing is performed (Kolmokorov-Sirnov 0.84 *p* < 2.2 E-16). In aseptic task procedure nursing the AHR is performed in a similar way at entry that at exit.

No AHR was detected in 42.5% of all the tasks, 37% of the ATPs, and 47.1% of the STPs for nursing (*p* = 0.0362), indicating that moment 2 was not respected in 37% of the mandatory situations. For the ATPs, the nurses performed better than the assistant nurses (35.9 vs. 51.6% of lack of compliance to M2; *p* < 0.001). The assistant nurses practicing STPs did not perform AHR at all in 52% of these nursing tasks. The housekeeping workers only performed STPs, and AHR was not detected in 40.6% of the tasks. Among the nursing tasks performed, those with lower lack of AHR execution was urinary catheter removal, blood catheter insertion, blood sample collection, intravenous medication, and blood cultures (Figure 1). Interestingly, some tasks that were considered STPs for nursing, such as penile case assessments and per-os medication, were associated with higher AHR performance. Figure 2 shows the distribution of AHR events among 16,416 nursing tasks, 12,759 classified STPs (in gray), and 2,853 ATPs (in black) during the nursing task. Hand hygiene is more frequently performed at the end of the task when STPs nursing is performed (Kolmokorov–Smirnov 0.84 *p* < 2.2 E-16). In ATPs nursing the AHR is performed in a similar way upon entry rather than upon exit. A multilevel analysis showed that the variables associated with AHR were the behavior of HWCs itself and the nature of the tasks. AHR was slightly performed more often by any of the HCWs when the task recorded was an ATP [ORa 1.08 (95% CI) (1.01–1.17) *p* = 0.036] (Supplementary Material).

## Discussion

Electronic hand hygiene systems have been developed not only to record but also to promote compliance. These systems have been designed to ensure that HCWs perform hand hygiene before approaching the patient's bedside (M1) and to issue an alert for HCWs to do so (18). Despite their advantages, newer technologies, at this time, are unable to differentiate the five moments for hand hygiene, but most of them can detect whether the hands of HCWs have been disinfected before touching a patient (moment 1) and before leaving the patient zone (similar to moments 3, 4, and 5). Nevertheless, the level of risk of infection is associated with specific steps in the care process and the relative importance of hand hygiene at each of the five moments in preventing microbial transmission and infection outcomes is still unknown (22). According to the WHO guidelines, it is assumed that “an ideal indicator of hand hygiene performance would reliably capture each moment requiring hand hygiene, even during complex care activities” (20). In this study, by merging the data obtained from the two systems, we successfully identified that in ATPs that are identified as high risk for cross-transmission of microbes to patients, no ARH was performed in 37% of the tasks. However, this lack of compliance may be underestimated. Among task for which we detect AHR it is possible that hand hygiene was performed after the aseptic procedure as reported in Figure 2, and consequently considered as inefficient. This is the main limitations of our study.

The lack of AHR among the nurses practicing ATPs is likely related to the fact that they have reduced the need for hand hygiene by wearing non-sterile gloves, which can make HCWs feel less exposed to dirt and microbes, and protected against blood-borne diseases (20). The overuse of gloves has been shown to be one major factor explaining poor hand hygiene compliance (23, 24). There is currently increasing scientific evidence that glove disinfection is as effective in preventing infections in experiments as in routine care, and that glove disinfection should be promoted (25, 26). Recently, Fehling et al. reported that allowing glove disinfection significantly improved hand disinfection, particularly at moment 2, and reduced the occurrence of severe infections considerably (27). The weakness of our system is that we detect the lack of AHR, but the technology available at this time is unable to detect AHR and whether it occurs precisely within the time of nursing. Only video capture is capable of doing it. A new generation of hand hygiene monitoring should evolve to dematerialized video system with real time data analysis and feedback.

In conclusion, assessing hand hygiene surveillance during complex care is feasible by combining different technologies. Although not perfectly responding to WHO requirements for an ideal indicator, this proof-of-concept study reveals that AHR is not performed in at least 37% of care situation for which it is mandatory. Focused interventions on the practice of moment 2 for hand hygiene should be quicky implemented and then evaluated.

## Data Availability Statement

The original contributions presented in the study are included in the article/Supplementary Material, further inquiries can be directed to the corresponding author/s.

## Ethics Statement

This study has been approved by the Ethical Review Board of IHU Mediterranée-Infection under N° 2016-018.

## Author Contributions

PB, JD, and SB conceived and designed the experiments. OF performed the experiments. OF, JG, and SB analyzed the data. OF, JG, and PB wrote the manuscript. OF, JD, and SB edited the manuscript. All authors contributed to the article and approved the submitted version.

## Funding

This study was funded by ANR-15-CE36-0004-01 and by ANR Investissements d'avenir, Mediterranée infection 10-IAHU-03.

## Conflict of Interest

PB owned part of the start-up company MedihandTrace SAS, which commercialized the electronic monitoring system. The remaining authors declare that the research was conducted in the absence of any commercial or financial relationships that could be construed as a potential conflict of interest.

## Publisher's Note

All claims expressed in this article are solely those of the authors and do not necessarily represent those of their affiliated organizations, or those of the publisher, the editors and the reviewers. Any product that may be evaluated in this article, or claim that may be made by its manufacturer, is not guaranteed or endorsed by the publisher.
