# Supplementary material for: Internet of Things to Explore Moment 2 of “WHO My Five Moments” for Hand Hygiene
Source: Front Digit Health. 2021 Oct 21;3:684746. doi: 10.3389/fdgth.2021.684746 (PMC8566730; doi:10.3389/fdgth.2021.684746)

In order to studies the link between the hand hygiene compliance and the type of nursing act, the following generalized linear mixed models were built:

$$M_0 : \ logit\left(P\left(Y_{ij} = 1\right)\right) = \beta_0$$

$$M_{0r} : \ logit\left(P\left(Y_{ij} = 1\right)\right) = \beta_0 + \ b_{0i} \qquad b_{0i} \sim N(0, \sigma_0^2)$$

$$M_1 : \ logit\left(P\left(Y_{ij} = 1\right)\right) = \beta_0 + \beta_1 X_{ij}$$

$$M_{1r} : \ logit\left(P\left(Y_{ij} = 1 \backslash b_{0i}\right)\right) = \beta_0 + \beta_1 X_{ij} + b_{0i} \qquad b_{0i} \sim N(0, \sigma_0^2)$$

Where Y is the hand hygiene compliance variable (Y=0 if no AHR use and Y=1 if AHR use) and X is the nursing act risk variable (X=0 if the nursing act is a SPP act and X=1 if the nursing act is a ATP act). The indice i represents the HCW level and j represents nursing care level, then Y_{ij} represents the hand hygiene compliance for the j th nursing act from the i th HCW.

The different models were compared between them, by several methods. The Akaike Inference Criterion (AIC) was used when models are not nested. For nested models, the comparison was made with likelihood ratio test corrected for comparison between two mixed models

$$M_0 \subset M_{0r} \subset M_{1r} \qquad M_0 \subset M_1 \subset M_{1r}$$

	M_0	M_{0r}	M_1	M_{1r}
Deviance	41580	38043.4	41421	38039.0
AIC	41582	38047.4	41425	38045.0

Comparison between M_0 and M_{0r} : LR = Dev(M0) – Dev(M0r) = 41580 – 38043.4 = 3536.6 (p < 0.0001)

Comparison between M_0 and M_1 : LR = Dev(M0) – Dev(M1) = 41580 – 41421 = 159 (p <0.0001)

Comparison between M_1 and M_{1r} : LR = Dev(M1) – Dev(M1r) = 41421 – 38039.0 = 3382 (p < 0.0001)

Comparison between M_{0r} and M_{1r}: LR = Dev(M0r) – Dev(M1r) = 38043.4 – 38039.0 = 4.4 (0.0359)

The selected model after comparison is the M_{1r} model. There is an effect of the nursing act risk variable on the hand hygiene compliance despite existence of individual variation.

Table 2 : Variable associated with hand hygiene

Multi-level logistic analysis (N=46 HCWs)	Nursing acts (N=30164, Ref= no AHR use)				
	N(%)	Null mixed model (M_{0r})		Full mixed model (M_{1r})	
		ORa (95% CI)	P	ORa (95% CI)	P
Fixed effect					
Individual	-	0.97 (0.67-1.3	0.881	0.96 (0.68-	0.837
adjusted OR		9)		1.36)	
Type of nursing	25633 (85.0)		-		-
acts	4531 (15.0)	-	-	1	0.036
SPP		-		1.08 (1.01-	
ATP	-		-	1.17)	-
Random effect	-	1.389	-		-
Interindividual		0.30		1.385	
variance	-		-	0.30	-
ICC	-	38047.4	-		-
Statistical model	-	38064.0	-	38045.0	-
AIC	-	-19021.7	-	38069.9	-
BIC		38043.4		-19019.5	
logLik				38039.0	
Deviance					

Medihandtrace ® Monitoring Solutions

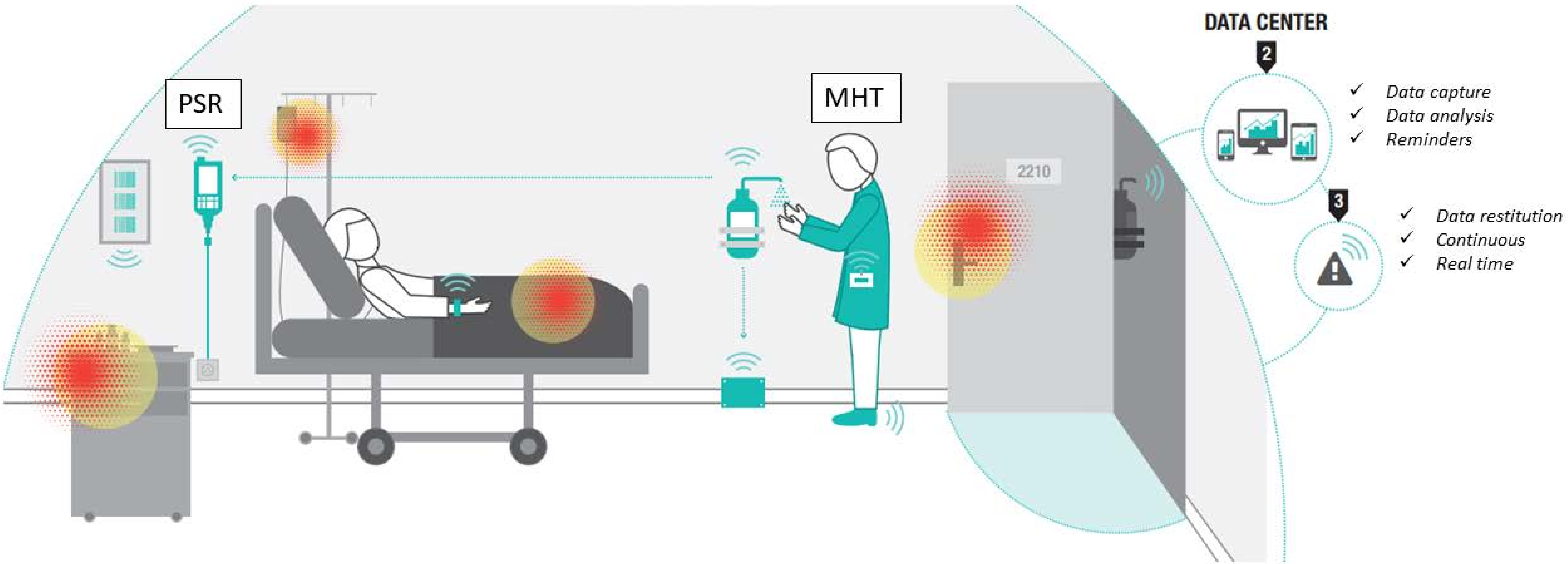

Supplement: Supplementary file 1 [file Data_Sheet_1.pdf]
